# Entérite lupique récidivante améliorée par Azathioprine

**DOI:** 10.11604/pamj.2015.20.215.5757

**Published:** 2015-03-10

**Authors:** Sameh Marzouk, Saida Garbaa, Yosra Cherif, Moez Jallouli, Fathi Bahri, Zouhir Bahloul

**Affiliations:** 1 Service de Médecine Interne, CHU Hédi Chaker, Sfax, Tunisie; 2 Service de Médecine Interne, CHU Farhat Hached, Sousse, Tunisie

**Keywords:** Entérite lupique, lupus érythémateux disséminé, Azathioprine, lupus enteritis, systemic lupus erythematosus, Azathioprine

## Abstract

Les manifestations gastro-intestinales observées au cours du lupus érythémateux systémique sont fréquentes et peuvent intéresser n'importe quel segment du tractus digestif. L'entérite lupique constitue l'une des manifestations responsable de douleurs abdominales. Son traitement est basé essentiellement sur les corticoïdes. Le recours aux immunosuppresseurs est réservé aux formes récidivantes ou en cas d’échec des corticoïdes. Nous rapportons une nouvelle observation d'entérite lupique récidivante améliorée par azathioprine. Il s'agissait d'une femme âgée de 30 ans chez laquelle le diagnostic du lupus a été retenu en 2004. Un an après, elle a présenté des douleurs abdominales, des vomissements et des diarrhées. Les explorations ont conclu à une entérite lupique après élimination de toute autre cause notamment infectieuse. Elle a été traitée par des corticoïdes à forte dose. Cependant à chaque tentative de dégression, elle présentait la même symptomatologie. En 2010 l'azathioprine a été associé permettant de juguler la maladie et de diminuer la corticothérapie.

## Introduction

Le lupus érythémateux systémique (LES) est une maladie auto-immune non spécifique d'organe, touchant préférentiellement la femme en âge de procréer et évoluant par poussées. Elle est caractérisée par des manifestations très polymorphes sur le plan clinique. L'entérite lupique constitue l'une des complications du lupus et une cause d'un syndrome douloureux abdominal [1]. Elle serait liée à une vascularite digestive. De ce fait elle nécessite une démarche diagnostique et thérapeutique rapide puisque la mortalité liée à une perforation ou à une hémorragie digestive n'est pas négligeable [2]. Les récidives sous corticoïdes sont fréquentes [3]. Dans ces cas l'association d'un immunosuppresseur s'avère nécessaire pour stabiliser la maladie. Nous rapportons une nouvelle observation d'entérite lupique récidivante qui a été traitée avec succès par l'azathioprine.

## Patient et observation

Mme I, S âgée de 30 ans, a présenté en mars 2004, dans les suites d'un accouchement de son premier bébé, une polyarthrite fixe touchant les épaules, les coudes, les genoux, les poignets, les métacarpophalangiennes et les interphalangiennes proximales associée à une fièvre chiffrée à 38,5^°^C, à une asthénie et à une éruption malaire photosensible. Il n'existait pas de symptomatologie digestive. A la biologie, on trouvait une vitesse de sédimentation à 80 mm à la première heure, une CRP négative, une leucopénie à 2600 cellules/mm^3^ avec une lymphopénie à 1070E/mm^3^, une protéinurie à 1,8 g/24 h sans hématurie ni insuffisance rénale. Les anticorps anti-nucléaires (AAN) étaient positifs à 1/320 avec des anti DNA et des anti Sm positifs. Les anticorps CCP étaient négatifs. Les anticorps cardiolipines étaient positifs. Le complément sérique était bas (C4 à 0,09g/l et C3 à 0,52g/l). L’électrocardiogramme, la radiographie pulmonaire et l’échographie cardiaque étaient normaux. La ponction biopsie rénale a conclu à une néphropathie lupique stade II de l'OMS. Au terme de ces examens on retenait le diagnostic de lupus érythémateux systémique et la patiente a été traitée par une corticothérapie à raison de 15mg 15mg/jour de Prednisone et de l'Hydrochloroquine avec une bonne évolution. Ce dernier a été arrêté suite à la survenue d'une urticaire. Un an plu tard, elle a présenté des douleurs abdominales diffuses associées à des diarrhées liquidiennes et à des vomissements dans un contexte fébrile. L'examen clinique trouvait un abdomen souple mais distendu et douloureux sans défense ni contracture. Il n'existait pas d'arthrite, ni de lésion cutanée.

**Figure 1:**
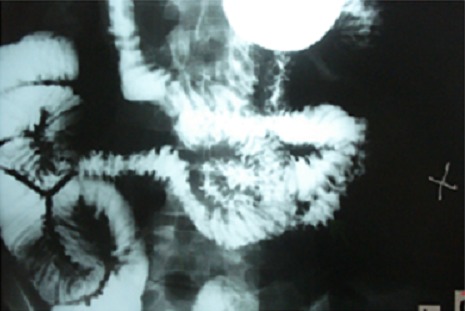
Transit du grêle: épaississement des anses jéjunales avec un aspect nodulaire

**Figure 2:**
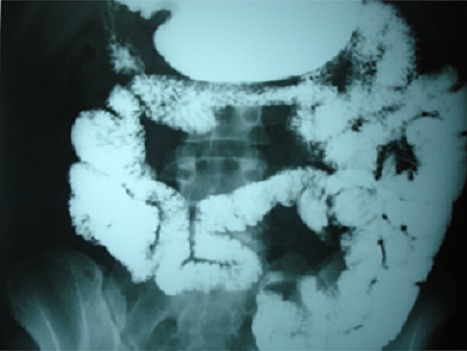
Amélioration après traitement

Les examens biologiques montraient une anémie à 9,1g/dl; une leucopénie à 2900 E/mm^3^; une VS à 109 mm H1, une CRP négative; des anticorps anti- DNA positifs à 455UI/m, des anti-Sm positifs et un complément sérique bas (CH50, C3 et C4). Les bilans hépatique, pancréatique, rénal et thyroïdien étaient normaux. L'enquête infectieuse (sérologie typhoïde, Shigella, Yersinia, CMV, examen parasitologique des selles et coproculture) était négative. L'Abdomen sans préparation a montré des niveaux hydroaériques. Le scanner abdominal a montré une splénomégalie, une rectite aigue et une distension liquidienne du grêle avec un rehaussement pariétal des anses iléales. La colonoscopie a montré une rectite basse avec à l'histologie une rectite oedémateuse non spécifique. La fibroscopie a conclu à une gastrite. Le transit du grêle a objectivé une atteinte étendue jéjunale avec épaississement et un aspect nodulaire (Figure 1). Le diagnostic d'une entérite lupique a été retenu. La patiente a été traitée par une corticothérapie à raison de 1mg/kg/j entrainant une amélioration clinique avec disparition de la fièvre et des signes digestifs. Cependant à chaque tentative de dégression à une dose inférieure à 30 mg/j de Prednisone, on assistait à une récidive de la même symptomatologie nécessitant l'augmentation des doses voire même le recours à des bolus de Solumédrol de 1g/j pendant 3 jours. En mai 2010, après plusieurs poussées digestives, et alors qu'elle était sous 30 mg/j de prednisone, l'azathioprine a été associé à la dose de 2mg/kg/j. L’évolution était favorable sans récidive des manifestations digestives permettant de diminuer les doses des corticoides (Figure 2). La patiente est actuellement sous 10 mg de corticoïdes sans nouvelle poussée lupique avec un recul de 15 mois.

## Discussion

Les manifestations gastro-intestinales observées au cours du LES sont fréquentes, de l'ordre de 50%. Elles peuvent intéresser n'importe quel segment du tractus digestif. Les lésions muqueuses orales, la dysmotilité oesophagienne, l'entéropathie exsudative et la pancréatite constituent les manifestations les plus fréquentes [4]. L'entérite lupique est une complication rare au cours du LES. Sa prévalence est estimée entre 0,2 et 2%. Cependant elle représente une cause fréquente des douleurs abdominales selon certaines séries, signalée dans 45 à 79% des cas [5, 6]. Dans notre série, elle représente la première observation parmi une série de 148 LES. Le tableau clinique d'une entérite lupique est non spécifique. Il associe souvent des douleurs abdominales aigues, des vomissements, des diarrhées, parfois même un syndrome occlusif et des signes d'irritation péritonéale [2, 7]. Notre patiente présentait, des douleurs abdominales, des diarrhées, des vomissements voire même un syndrome sub-occlusif. Sur le plan physiopathologique, l'entérite lupique est la conséquence d'une vascularite des petits vaisseaux (artérioles et veinules) associant une atrophie, une dégénérescence de la média, une nécrose fibrinoïde, une thrombose ancienne, une phlébite et une infiltration de la lamina propria par des monocytes [7, 8]. Les complications de l'entérite sont rares. Elles sont liées à une hémorragie massive ou à une perforation digestive pouvant aggraver le pronostic vital [7, 9]. Les examens radiologiques sont d'un grand apport pour le diagnostic. L'atteinte est le plus souvent jéjunale ou iléale [5, 6, 9]. Notre malade présente effectivement une atteinte jéjunale.

Le scanner abdominal montre un épaississement de la paroi intestinale, des images en cocarde, un œdème mésentérique et parfois une ascite. Cependant ces lésions ne sont pas spécifiques, puisqu'elles peuvent être vues dans d'autres maladies digestives [6]. Le scanner permet ainsi d’éliminer d'autres causes des douleurs abdominales comme la pancréatite. Ainsi le diagnostic d'entérite lupique reste un diagnostic d'exclusion et nécessite dans tous les cas un bilan complet afin d’éliminer les autres causes de douleurs abdominales et les causes infectieuses [2, 9]. Les endoscopies digestives, lorsqu'elles sont pratiquées, retrouvent habituellement une paroi digestive très oedématiée et même des ulcérations [2]. Dans notre observation, la colonoscopie a mis en évidence un aspect oedématié mais l'histologie n'a pas montré de lésion de vascularite. Le traitement initial de l'entérite lupique est basé sur des fortes doses de corticoïdes entrainant une bonne réponse. Cependant, des rechutes ne sont pas rares, même chez les patients qui présentent une bonne réponse initiale à ce traitement [3, 7]. Kim et coll [10] ont montré qu'il n'existait pas de différences significatives concernant les données épidémiologiques, cliniques ou biologiques y compris le profil des autoanticorps et le SLEDAI, entre les formes d'entérite lupique aves ou sans récidive. Cependant, il a noté que chez les patients sans récidive, la dose cumulative de prednisolone et la durée du traitement ont été significativement plus élevées par rapport aux patients avec récidive. Le recours à d'autres thérapeutiques s'avère parfois nécessaire en cas d’échec des corticoïdes ou en cas de cortico-dépendance. Grimbacher [8] a rapporté une rémission complète d'une entérite lupique récidivante par des perfusions de cyclophosphamide. D'autres thérapeutiques ont prouvé leur efficacité comme le mycofénolate mofétil, l'azathioprine, le tarcolimus et le Rituximab [3, 11, 12]. Notre cas se caractérise par les récidives fréquentes à des doses élevées de corticoïdes nécessitant l'adjonction de l'azathioprine ce qui a permis de stabiliser la maladie.

## Conclusion

L'entérite lupique est une manifestation rare mais constitue l'une des principales causes des douleurs abdominales aigues au cours du LES. Son diagnostic repose sur des éléments cliniques, biologiques et radiologiques après élimination des autres causes des douleurs abdominales. Sont traitement est basé surtout sur les corticoïdes. Cependant les récidives sont fréquentes. L'Azathioprine constitue une alternative thérapeutique permettant de juguler la maladie.
